# Positive attributes in elderly people with different degrees of depression: a study based on network analysis

**DOI:** 10.1186/s41155-022-00244-w

**Published:** 2023-01-10

**Authors:** Sabrina Braga dos Santos, Wagner de Lara Machado, Liana Lisboa Fernandez, Analuiza Camozatto de Pádua, Sofia Hoffmann, Prisla Ücker Calvetti, Bruno Luis Schaab, Caroline Tozzi Reppold

**Affiliations:** 1 Department of Speech Therapy at Centro Universitário Metodista — IPA, Cel Joaquim Pedro Salgado, 80, Bairro Rio Branco, Porto Alegre, Rio Grande do Sul 90420-060 Brazil; 2 Graduate Program in Psychology at Pontifícia Universidade Católica do Rio Grande do Sul — PUCRS, Health Sciences School, Av. Ipiranga, 6681, 9° andar, sala 930, Partenon, Porto Alegre, Rio Grande do Sul 90619-900 Brazil; 3 Department of Basic Health Sciences at Universidade Federal de Ciências da Saúde de Porto alegre — UFCSPA, Rua Sarmento Leite, 245, Porto Alegre, Rio Grande do Sul 90050-170 Brazil; 4 Department of Medical Clinical at Universidade Federal de Ciências da Saúde de Porto Alegre — UFCSPA, Rua Sarmento Leite, 245, Porto Alegre, Rio Grande do Sul 90050-170 Brazil; 5 Psychology at Universidade Federal de Ciências da Saúde de Porto Alegre — UFCSPA, Rua Sarmento Leite, 245, Porto Alegre, Rio Grande do Sul 90050-170 Brazil; 6 Psychological Assessment Laboratory at Universidade Federal de Ciências da Saúde de Porto Alegre — UFCSPA, Rua Sarmento Leite, 245, Porto Alegre, Rio Grande do Sul 90050-170 Brazil; 7 Psychological Assessment Laboratory in Department of Psychology at Universidade Federal de Ciências da Saúde de Porto Alegre — UFCSPA, Rua Sarmento Leite, 245, Porto Alegre, Rio Grande do Sul 90050-170 Brazil

**Keywords:** Aging, Depression, Well-being, Positive Psychology, Network analysis

## Abstract

**Introduction:**

Depression in aging may lead to loss of autonomy and worsening of comorbidities. Understanding how positive attributes contribute to healthier and happier aging has been one of the purposes of Positive Psychology. However, the literature still lacks studies that evaluate how depression in the elderly is related to constructs considered positive.

**Objective:**

The present study aimed comparing scores of constructs of spiritual well-being, social support, self-esteem, life satisfaction, affection, optimism, and hope in the elderly with minimal, mild, moderate, and severe depression and healthy controls in order to investigate possible indirect and mediated relationships between positive constructs and depression.

**Methods:**

A cross-sectional study was conducted with elderly, 62 of whom were diagnosed with different severity of Major Depression (DSM-V) (minimum, mild, moderate, and severe according to the Beck Depression Inventory — BDI) and 66 healthy controls matched by age, sex and schooling. The instruments used were adapted and validated versions of the Spirituality Self-Rating Scale, the Rosenberg Self-Esteem Scale, the Medical Outcomes Social Scale of Support, the Life Satisfaction Scale, the Positive and Negative Affect Schedule, the Revised Life Orientation Test, and the Adult Dispositional Hope Scale. After comparing the means of scores between groups, an analysis of normalized partial association networks was performed to investigate the direct and mediated relationships between depression and other evaluated constructs.

**Results:**

Scores of spiritual well-being, social support, self-esteem, life satisfaction, positive affect, optimism, negative affects, and hope differed significantly between the control group and the degrees of depression (*p* < 0.001). The analysis of normalized partial association networks has shown that the relations of depression with the constructs of life satisfaction, self-esteem, and social support are mediated, while the constructs of dispositional hope, positive affect, spiritual well-being, and optimism are indirectly related to depression. The social class was also positively related to depression.

**Conclusion:**

Depression in different degrees is associated with a reduction in the scores of instruments that evaluate positive attributes. The constructs directly associated with depression are spiritual well-being, optimism, positive affect, and dispositional hope. The others had mediated relationship. These results may contribute to the planning of future interventions for the prevention of depression among the elderly.

Nearly half of the elderly in Brazil (48.9%) presents more than one chronic illness, and one of the most serious is depression (Hellwig et al., 2016). One of the most common mood disorders, especially among the elderly, is major depressive disorder — MDD (Hellwig et al., 2016). The main diagnostic criteria are depressed mood and loss of interest and/or pleasure, as well as changes in sleep and/or appetite, agitation or slowness, fatigue, feelings of worthlessness or guilt, reduced thinking, and nihilism (DSM-5, 2013). Stressful events, chemical, biological, and social changes due to aging, imply changes that are risks for MDD, and this can lead to a reduction in quality of life, loss of autonomy, and aggravation of previous comorbidities (Galatzer-Levy & Bonanno, 2014; Marques et al., 2017).

Studies conducted with this clinical population investigate risk factors and the damages associated with the disease. However, Positive Psychology is an area that seeks to address positive characteristics of the individual, for health and well-being, expanding the focus of previous studies, often focused on suffering, clinical losses, and pathologies (Seligman, 2011). Some of the constructs most commonly investigated in Positive Psychology, including in clinical trials, are spirituality, social support, self-esteem, and life satisfaction along with affections in subjective well-being, optimism, and hope (Snyder et al., 2011).

International researches conducted with the elderly population indicate that there are evidences of an inverse and significant relationship between depression and spirituality (Abu-Raiya et al., 2016; Bamonti et al., 2014; Bashir et al., 2016), self-esteem (Orth et al., 2016), positive affects (Proyer et al., 2014), optimism (Ho et al., 2014; Niklasson et al., 2017), hope (Mirbagher et al., 2016; Mozooni et al., 2017), social support (Dangel & Webb, 2017), and life satisfaction (Adams et al., 2015). This results show the importance of the improved health developmental in elderly in contrast to focus in pathological development.

However, in the national context, such research is scarce. In addition, there are few international studies that explore the differences in Positive Psychology construct scores in elderly individuals with different degrees of depression. Considering this, the objective of the present research has compared constructs scores of spiritual well-being, social support, self-esteem, life satisfaction, affection, optimism, and hope in the elderly with minimal, mild, and moderate depression and healthy controls, order to investigate possible indirect and mediated relationships between positive constructs and depression.

## Methods

This was a cross-sectional study. The subjects were matched by age and sex. The sample was previously calculated in the program WinPepi version 11.43, based on the studies of Moreno et al. (2010) and Hernandez et al. (2009), considering a significance level of 5%, power 90%, and a standardized effect size of at least 0.6 standard deviations between groups regarding life satisfaction, resulting in at least 60 individuals with major depression (MD) and 62 healthy individuals in a control group. Participants were included for convenience in the study, resulting in a total of 128 subjects, 66 of whom were in the control group. The research was carried out following the ethical recommendations of research (National Health Council, resolution 466/12), guaranteeing the anonymity of data and was previously approved by a research ethics committee under opinion of number 1.046.803.

The instruments were applied by a trained team in clinical group of the outpatient clinic of the hospital in the metropolitan region of Porto Alegre and the control group in a physical activity group at a university. In the clinical group, 62 individuals who were being followed up in a mental health outpatient clinic of a hospital in a capital of southern Brazil were included. The inclusion criteria for this group were the diagnosis of MDD according to DSM-V criteria (DSM-5, 2013). The Beck Depression Inventory II (BDI-II) was used to assess the severity of the depressive episode (Gorenstein et al., 2011), considering the following categories: 0 to 13 points — minimum depression; 14–19 points — mild depression; 20 to 28 points — moderate depression; and 29–63 points — severe depression. Patients with dementia or diagnosis of another mental disorder were excluded from this group. These selection criteria were evaluated by doctors with clinical experience in evaluation of mental disorders. The selection of the participants in the control group was among the elderly who performed physical activity in an active aging group of a capital of the south of Brazil. All of them were healthy and functionally independent. The GDS (Geriatric Depression Scale) (Paradela et al., 2005; Sheikh & Yesavage, 1986) and the mental state mini-exam (MSME) (Bertolucci et al., 1994; Folstein et al., 1975) were applied to rule out suspected cases of depression and cognitive impairment, respectively. In order to evaluate the constructs of Positive Psychology in both groups, the following instruments were used: the Spirituality Self-Rating Scale — SSRS to evaluate spirituality in its adaptation to Brazilian Portuguese developed by Gonçalves and Pillon (2009); the medical outcomes study social support scale — MOS in adapted version to the Brazilian Portuguese (Griep et al., 2005) to evaluate social support; the Rosenberg Self-Esteem Scale — RSS to assess self-esteem in its Brazilian version by Hutz and Zanon (2011); the Life Satisfaction Scale — LSE in validated version for Brazilian Portuguese (Zanon et al., 2014) to evaluate life satisfaction; the PANAS — Positive and Negative Affect Schedule — in its Brazilian version (Zanon et al., 2013) to evaluate affections; the Revised Life Orientation Test (LOT-R) to evaluate the optimism in the Brazilian version (Bastianello et al., 2014); and the Adult Dispositional Hope Scale (ADHS) to evaluate hope in the Brazilian version (Pacico et al., 2013). All the instruments were used in their validated versions for the application in Brazil and have good indicators of validity and reliability. The order of application of the tests was random, in order to avoid bias in the responses.

### Statistical analysis

Quantitative data processing was performed using SPSS software, version 22.0. After obtaining the total scores of the dimensions were verified the assumptions of normality, homoscedasticity, and sphericity. The Mann-Whitney *U*-test was used to compare age between the case and control groups; the chi-square association test was used to compare the other demographic data between the groups. The Kruskal-Wallis and Wilcoxon-Mann-Whitney tests (test *U*) with significance adjusted by the Bonferroni test were applied to compare the scores of the PP scales between the depressed and healthy control groups. The level of significance was set at 5% (*p* ≤ 0.05).

Subsequently, regularized partial regression network analyses (Lauritzen, 1996) were conducted through the qgraph (Epskamp et al., 2012) package of statistical software R. The regularized partial correlation analyses aim to investigate the conditioned relations between depression and PP construct scores.

In this technique, each pair of variables are regressed, controlling the effect of the other variables analyzed. In order to avoid overadjustment of the model to the data, a penalty hyperparameter is used by means of the graphical least absolutes shrinkage and selection operator (GLASSO) method (Friedman et al., 2010) that zeroes edges with magnitudes close to zero. The choice of the best model is given by the extended Bayesian criterion (EBIC) index to generate the least residual graph (Foygel & Drton, 2010). Finally, the shortest pathways between the investigated variables were estimated in order to determine if they have direct or mediated relations in the model (Opsahl et al., 2010). Regularized partial correlations can be interpreted as regression betas, with normalized partial correlation coefficients being standardized and having cutoff points of 0.1 for weak, 0.3 for moderate, and 0.5 for strong correlation between variables. These values are due to the rigid control of the influences between variables in the association between them (Opsahl et al., 2010).

In order to represent the regularized partial correlations, a graph indicating the partial correlations (or Markov Random Field; Lauritzen, 1996), that is, pairwise associations after the statistical control of the other variables of the model (i.e. conditionals), was generated. In this technique, an adjacency matrix (i.e., regularized partial correlation matrix) is represented by means of a graphic object. In this graph, the variables are represented by vertices (or circles) and the relations between the variables as edges (or lines). The intensity of the edges of the graph represents the magnitude of these associations, while their color, red or green, represents the direction (negative or positive, respectively) of the associations. The graph also has the application of a positioning algorithm (Fruchterman & Reingold, 1991), in which variables are approximated or expelled according to their association. The variables represented in the center of the graph have a greater number of associations (Machado et al., 2015). In this analysis, the “negative affects” construct was excluded from the analysis of correlation, association, network, and centrality due to its proximity to depression.

## Results

Table 1 presents the comparison of demographic data between the groups of healthy individuals (controls) and the group with mild, moderate, and severe degrees of depression. The analysis of these data in the table indicates the pairing of groups by age, sex, and schooling.

**Table 1:** Characteristics of depressive population in comparison to controls

	Controls *N* = 66	Depression *N* = 62	*p*
Age^a^	72.95 (7.63)^a^	71.91 (8.14)^b^	0.345
Sex^b^			
F	54 (81.8)	48 (75.8)	0.405
*Scholarity*^*b*^			
*Never studied*	*0 (0)*	*6 (9.7)*	*0.076*
Until first degree	53 (80.3)	45 (72.6)	
Up to second degree	7 (10.6)	5 (8.1)	
Higher and/or postgraduate	6 (9.1)	6 (9.7)	
Social class^a^			
A	4 (6.1)	0 (0)	< 0.001
B	20 (30.3)	1 (20)	
C	39 (59.1)	43 (69.4)	
D	3 (4.5)	15 (24.2)	
E	0 (0)	3 (4.8)	
Use of antidepressants^a^			
Sim	7 (10.6)	54 (87.1)	< 0.001

^a^Mann-Whitney*U*-test, results presented as mean and standard deviation

^b^Chi-square, results presented in *n* (%)

Comparison of scores on the scales of spiritual well-being, social support, self-esteem, life satisfaction, positive affect, negative affect, optimism, and hope among healthy elderly individuals with mild, moderate, and severe degrees of depression was demonstrated in Table 2. There was a significant difference in the comparison of the scores of all PP constructs between the groups. Multiple comparisons made possible an analysis between groups, as described below.

**Table 2:** Comparison of scores on the spiritual well-being, social support, self-esteem, life satisfaction, positive affects, negative affects, optimism, and hope between individuals with different degrees of depression (BDI-II) and the control group

	Controls *N* = 66	Minimum (0–13) *N* = 8	Light (14–9) *N* = 11	Moderated (20–28) *N* = 16	Severe (29–63) *N* = 27	*p*	H
Spiritual well-being	38.0 (16–48)^a^	33.5 (15–38)^b^	24.5 (17–31)^bc^	17.0 (11–33)^c^	13.0 (1–27)^d^	< 0.001	93.97
Peace	13.0 (5–16)^a^	10.5 (3–14)^b^	7.5 (6–9)^c^	5.0 (3–11)^c^	3.0 (0–8)^d^	< 0.001	92.59
Sense	13.0 (8–16)^a^	11.0 (4–13)^b^	6.5 (5–10)^bc^	6.0 (4–11)^c^	4.0 (0–9)^d^	< 0.001	95.4
Faith	13.0 (3–16)^a^	12.0 (7–12)^b^	9.5 (6–13)^bc^	7.0 (2–12)^c^	6.0 (0–12)^c^	< 0.001	76.37
Social support	100 (41–100)^a^	76 (33–100)^ab^	54 (20–100)^bc^	40 (20–100)^bc^	44 (20–100)^ec^	< 0.001	76.93
Material	100 (20–100)^a^	80 (20–100)^b^	70 (20–100)^bc^	60 (20–100)^cd^	60 (20–100)^bcd^	< 0.001	56.76
Affective	100 (46–100)^a^	90 (40–100)^b^	60 (20–100)^bc^	40 (20–100)^c^	40 (20–100)^c^	< 0.001	86.03
Emotional	100 (40–100)^a^	90 (25–100)^ab^	45 (20–100)^bc^	40 (20–100)^bc^	40 (20–100)^c^	< 0.001	78.06
Information	100 (40–100)^a^	90 (20–100)^ab^	40 (20–100)^bc^	40 (20–100)^bc^	40 (20–100)^c^	< 0.001	78.41
Positive social interaction	100 (40–100)^a^	60 (40–100)^b^	40 (20–100)^bc^	40 (20–100)^bc^	20 (20–100)^c^	< 0.001	83.59
Self-esteem	39 (30–40)^a^	36.5 (17–40)^b^	22 (16–31)^c^	20 (13–33)^c^	14 (10–26)^d^	< 0.001	95.97
Life satisfaction	32 (12–35)^a^	28 (14–32)^b^	23 (19–28)^bc^	20 (9–29)^cd^	15 (5–24)^d^	< 0.001	87.82
Positive affects	38 (27–48)^a^	31 (19–47)^a^	17 (11–28)^b^	15 (10–30)^bc^	13 (10–24)^bc^	< 0.001	92.88
Negative affects	11 (10–41)^a^	14 (10–24)^ab^	18.5 (16–24)^bc^	20 (13–35)^bcd^	26 (15–35)^d^	< 0.001	75.93
Optimism	24 (9–30)^a^	24 (10–27)^ab^	17 (7–24)^bc^	11 (3–20)^c^	6 (0–15)^d^	< 0.001	83.47
Hope	38 (24–40)^a^	34.5 (14–39)^ab^	24 (20–31)^bc^	20 (12–29)^c^	16 (8–23)^d^	< 0.001	94.25

^*a,b,c,d^Different letters indicate significant difference (Kruskal-Wallis with post hoc *U* of Mann-Whitney with Bonferroni significance correction)

^**^Values presented in median and amplitude — m (min-max)

^***^H = Kruskal-Wallis test

Spiritual well-being construct is evaluated in three different factors: “peace,” “meaning,” and “faith.” In the overall spiritual well-being score, multiple comparisons between the evaluated groups showed higher scores in the control group compared to all degrees of depression. The group with indicators of minimal depression had scores higher than the group of moderate depression and the group of severe depression, and the latter presented reduced scores in comparison with all the other groups. When peace, sense, and faith were evaluated in isolation, the control group also showed higher scores than the other groups. In relation to faith, the scores of moderate depression did not differ from the severe one. The minimal depression differed from the mild only in “peace.”

Regarding the evaluation of the overall social support construct score, the results indicated that the control and minimal depression groups did not differ, but the control differed from the other clinical groups. When assessing isolation factors, it was observed that the same occurred in relation to the dimensions of social emotional support and information. In the dimensions of positive social interaction, affective and material support, the control group presented the highest scores, differing from all other groups. 

Regarding the self-esteem construct, the results indicated that the control group had a significantly higher mean score than the other groups. The comparison of the clinical groups showed differences between them, except between the mild and moderate depression groups, and the group with severe depression had lower mean scores than all the others. In the life satisfaction scores, the control group had averages higher than the others. The means of the minimal depression group differed from the moderate depression and severe depression groups, but did not differ from the mild depression group, and the latter group also differed from the severely depressed group.

The affections had positive and negative affect scores added separately, and the averages were distributed as follows: positive affections had higher mean values in the control and minimal depression groups compared to the other clinical groups, but the control and minimal depression groups did not differ. On the other hand, the negative affects had lower scores in the control group and minimal depression in relation to the other clinical groups, and the severe depression group presented the highest averages in relation to the means of the control, minimal depression, and mild depression groups. Optimism and hope presented higher means in the control and minimal depression groups compared to the moderate and severe depression groups, and control and minimal did not differ, and also, there was no difference between minimal and mild depression groups. The severe depression group had smaller scores than the other groups.

**Fig. 1:**
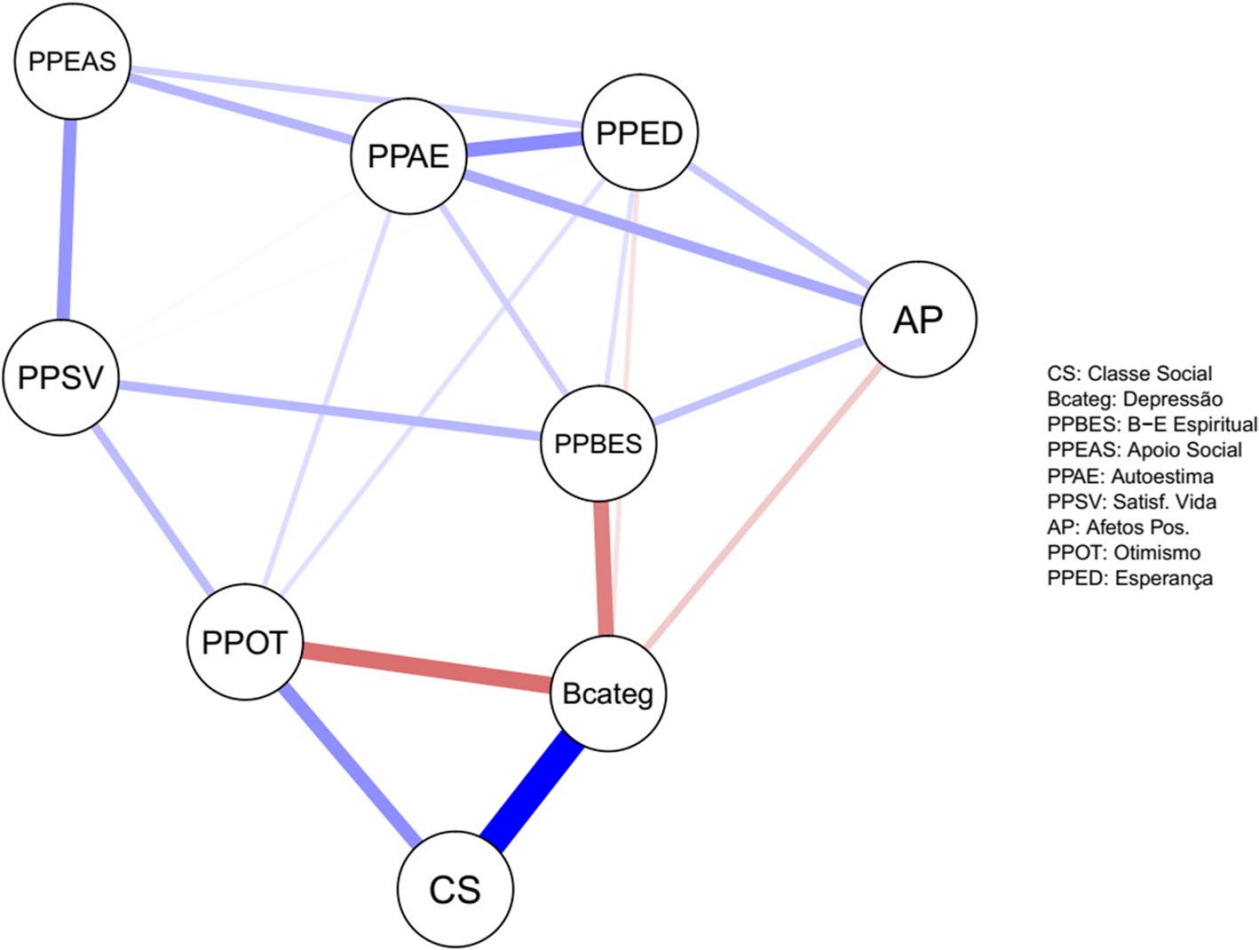
Regularized partial network — Glasso method between Positive Psychology constructs and indicators of depression. CS, social class; Bcateg, depression scores; PPBES, spiritual well-being; PPEAS, social support; PPAE, self-esteem; PPSV, life satisfaction; AP, positive affects; PPOT, optimism; PPED, dispositional hope. The blue color assumed to be positive vs. red color negative relationship and the width of the lines (assumed to correspond to strength of the relationship)

**Fig. 2:**
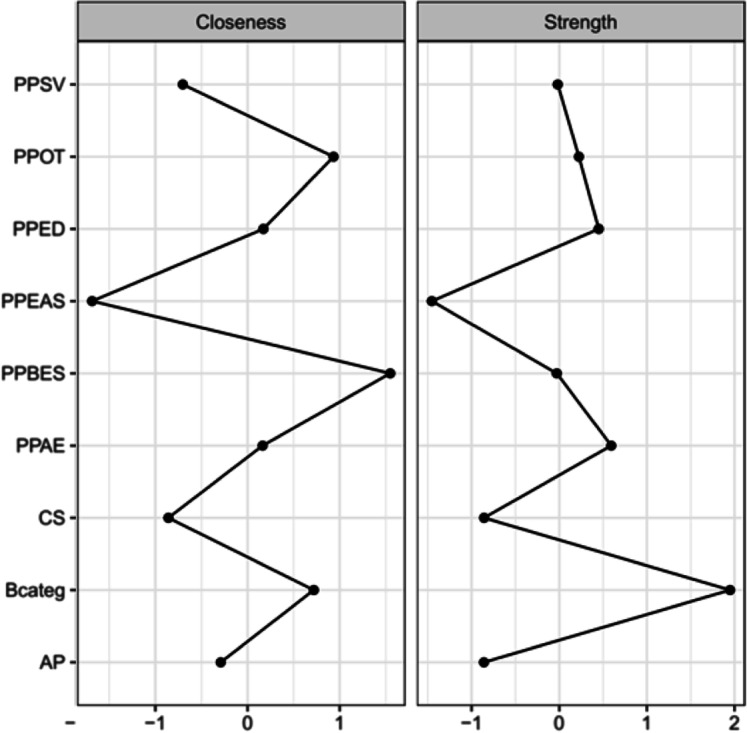
Measures of centrality between the constructs of Positive Psychology and the indicators of depression. Legend: Measures of values standardized with zero mean, “closeness” represents the variables with the highest number of weighted connections, and “expected influence” represents the variables with the greatest positive influence over the others

In Figs. 1 and 2, the network of partial correlations is represented by the network analysis, that is, the peer relations after controlling for the effects of the other investigated variables. In comparison with the bivariate correlations, it was observed that this network maintains only those relations less dependent and more stable in this system. It was possible to emphasize that the variables social class, optimism and spiritual well-being had greater association with depression. Still, the variables social-support, self-esteem, life satisfaction, and dispositional hope had associations mediated by other constructs with depression. Life satisfaction had relation to depression mediated by optimism, while social support had its relation to depression mediated by life satisfaction and optimism. Self-esteem had association with depression mediated by spiritual well-being, just like the dispositional hope. Depression also had a direct and weak association with positive affect. In addition, the strong influence of the social level on the intensity of depression is highlighted.

Correlated partial correlations pointed to significant negative relationships between depression and spiritual well-being, as well as between depression and optimism. There was also a weaker negative relationship between depression and dispositional hope, positive affects, life satisfaction, and social support. Correlations also pointed to positive and significant relationships between depression and social class (Table 3). The shortest path model expressed in Table 3 below the dotted line indicates that the variables social class, spiritual well-being, positive affect, optimism, and dispositional, hope had a direct link to depression, while variables social support, self-esteem, and life satisfaction had mediated, i.e., indirect relationships with the outcome investigated (Table 3).

**Table 3:** Correlated partial correlations above the dotted line and minimum paths below the dotted line between depression, social class, spiritual well-being, social support, self-esteem, life satisfaction, positive affects, optimism, and dispositional hope

	1	2	3	4	5	6	7	8	9
	SC	Depression	SWB	SS	SE	LS	PA	OP	DH
SC	-	0.46	0.05	0.00	0.02	0.04	0.00	0.26	0.00
Depression	2, 1	-	−0.28	0.00	−0.05	−0.09	−0.17	−0.30	−0.14
SWB	3, 2, 1	3, 2	-	0.00	0.17	0.20	0.18	0.00	0.14
SS	4, 6, 8, 1	4, 6, 8, 2	4, 6, 3	-	0.20	0.25	0.02	0.05	0.17
SE	5, 8, 1	5, 3, 2	5, 3	5, 4	-	0.11	0.22	0.15	0.26
LS	6, 8, 1	6, 8, 2	6, 3	6, 4	6, 4, 5	-	0.05	0.19	0.10
PA	7, 2, 1	7, 2	7, 3	7, 5, 4	7, 5	7, 3, 6	-	0.00	0.18
OP	8, 1	l8, 2	8, 2, 3	8, 6, 4	l8, 5	8, 6	8, 2, 7	-	0.14
HD	9, 2, 1	9, 2	9, 3	9, 4	9, 5	9, 6	9, 7	9, 8	-

The analysis of the “closeness” variable represented that the variables optimism, spiritual well-being, and depression followed with greater influence in the system, presenting the highest weighted number of connections. These constructs produce or are more sensitive to changes in the status of other system variables, and their levels are more likely to radiate changes in a greater number of variables. The “strength” variable indicated that the depression variable has the highest magnitude relationships in the investigated system, followed by the variables of dispositional hope and self-esteem, in order to indicate that changes in the status of this variable have a strong impact on neighboring variables.

## Discussion

The present research aimed to compare the constructs of spiritual well-being, social support, self-esteem, life satisfaction, affection, optimism, and hope among elderly people with degrees of depression severities and healthy elderly controls, as well as to investigate possible direct and mediated relationships between positive constructs and depression. The results indicated that healthy elderly with different depression degrees differs significantly in several of the evaluated constructs. Individuals with depressed mood had not only an increase in negative emotions scores but also a reduction of positive construct scores, especially in the severe degree of the disease. On spiritual well-being, spirituality scores fall progressively with the severity of the disease in the present study. This negative correlation was also evidenced in the analysis of correlation and networks of this study, this association being directly existent, that is, without mediating other factors, evidencing the great importance of spiritual well-being in depression. Viewed as a closeness factor, spiritual well-being has great influence and generates changes in other constructs and in depression with a greater probability of intervention.

The association of spirituality and depression was previously explored in clinical trials, which found a benefit and a protective association of spirituality in aging, as well as a better quality of life and reduction of depression scores (Abu-Raiya et al., 2016; Elham et al., 2015), which is in agreement with the findings of the present study, in which the degrees of depression are lower in individuals with greater spirituality. This association in relation to degrees of depression was previously envisaged in the study by Bamonti et al. (2014), whose results showed a significant relationship between depression and spirituality, even citing it as a factor to be taken into consideration in the therapy of these elderly individuals for an improvement of the sense of life and, consequently, reduction of the levels of depression (Bamonti et al., 2014). The study by Bashir et al. (2016) found a negative association between depression and spiritual well-being through the same instruments used in the present study. However, mediating factors have not been previously explored, and the relationship of how the constructs influence depression or between them is unheard of in this study (Reis & Menezes, 2017). Considering the network analysis, the strength of the association between spirituality and depression in the present study and the benefits observed in previous studies suggest that spirituality is central in the life of the elderly and a promising construct when approached with the elderly with MDD (Kim et al., 2013; Kleiman et al., 2013).

Another issue considered central in the life of the elderly is social support, which also presented a negative relation to depression but with an indirect relationship and mediated by spiritual well-being, self-esteem, life satisfaction, and optimism. It did not present a position of great importance in the analysis of networks or of centrality, which leads one to believe that it plays a noncentral role for the depressive individual. Corroborating with the findings described on this construct, studies suggest that the impact of stress on depression can be reduced in individuals with greater social support, avoiding the feeling of loneliness (Faramarzi et al., 2015; Wang et al., 2014). However, because of depressive moods, individuals do not benefit from the available social support while remaining lonely. Previous studies find it difficult to establish the causality of this relationship, and some authors have previously indicated that this association may be indirect, mediated by cognitive and social factors (Liu et al., 2016; Wang et al., 2014; Wicke et al., 2014), corroborating findings of the present study. The study by Dangel and Webb (2017) found a mediated association of social support with psychological distress, while spirituality had a direct relationship. The Gallardo-Peralta (2017) also provides evidence that religiosity directly influences social support in the elderly through support from the congregation and satisfaction with the social relations resulting from this experience. The same was evident in the study by Bailly et al. (2018), who followed older people for 5 years and observed higher levels of social support when they had higher levels of spirituality. The various causal mediations of the relationship of social support to depression often reduce the possible relationship between them, thus creating a different impact for each individual (Smith et al., 2015).

As well as social support, self-esteem presented an association with depression in the present study and a correlation mediated by spiritual well-being. Although there is a relationship between self-esteem and depression, the longitudinal study by Gana et al. (2015) observed reciprocal effects between self-esteem and depression and concluded that both follow parallel trajectories during aging, but there is no relationship between them over the years, that is, one does not influence the other. It also states that self-esteem is a persistent individual trait with more stability than depressive mood, and one is neither necessary nor sufficient for the existence of the other (Gana et al., 2015). Self-esteem related with spirituality has been previously studied by Papazisis et al. (2013), that is, a strong religious belief is related to increased self-esteem as well as reduced stress and depression. The study, however, is not with the elderly, and large studies with the elderly have not been found in the literature for investigation of this relationship.

Other factors are taken into account in the association between self-esteem and depression. Among them, the presence of other emotional deregulators as an aggravating factor, such as the absence of social support (Marroquín, 2011) and the reduction in full attention (Bajaj et al., 2016), suggests a decrease in self-esteem and an increase in depression. Depression and self-esteem are genetically related (Franz et al., 2012), mainly by the oxytocin receptor gene, which is linked to the domains of self-esteem, optimism, and depression. Physiological and neural factors such as hippocampal volume, asymmetry of the prefrontal cortex, and cortisol reactivity are also studied as variables that influence both self-esteem and depression (Gana et al., 2015).

Depression is associated with worse life satisfaction, however, mediated by optimism. The association between life satisfaction and depression was reported in a previous study. However, this association was mediated by higher levels of disability and greater number of medical comorbidities (Subramaniam et al., 2016). In the literature, life satisfaction in the elderly is especially related to geriatric syndromes, which often result in reduced life satisfaction and self-esteem and increased depression (Yang et al., 2015), corroborating the present study. Life satisfaction is not directly related to depression but rather to mediators that result in an increase in depression scores. Social support was mediated by life satisfaction and mediated by optimism and spiritual well-being in its relation to depression. The study by Adams et al. (2015) presented these different relationships and points to social support as mediator of the relationship between life satisfaction and depression. The study by Roh et al. (2013) evidenced a mediation of spirituality in the relationship between life satisfaction and depression, corroborating the findings of the present study. Relationships are still little explored, but life satisfaction is indeed influenced by other constructs in its relation to depression, and more studies are needed to better understand these relationships.

Already with an association with depression, but without any mediation, positive affects also present averages negatively related to depression. With regard to well-being and affections, research shows benefits for the elderly in targeted interventions, aiding in the improvement of depressive symptomatology (Friedman et al., 2017; Proyer et al., 2014; Sutipan et al., 2017). The association between increased scores of positive affects and reduction of negative affects with depression was previously observed in the study by Hu and Gruber (2009). Degrees of depression have not been previously studied, but this change in affections in depression is expected, since depression itself has, in its definition, negative affects, such as guilt and worthlessness (DSM-5).

Another factor commonly associated with the diagnosis of depression is the loss of optimism, which in the present study has shown a strong and direct negative association with depression, as is spiritual well-being. Optimism and degrees of depression were not reported in earlier studies, but in cross-sectional studies, low optimism scores were associated with depression, including the risk of long-term depression (Giardini et al., 2017; Niklasson et al., 2017). Optimism is also studied as a predictor of better prognosis in depression (Ji et al., 2017), and the efficacy of interventions in previous clinical trials has been demonstrated (Gitlin et al., 2017; Ho et al., 2014). Optimism also appears as a measure of centrality in the present study, as well as a variable that can be predictor of outcomes as a mediator of relationships among other constructs and depression, with many possible influences in relation to depression. The only variable that seems to be a partial mediator of the relationship between optimism and depression is social class, which, even though it was not a construct of positive psychology, was kept in the analysis by directly influencing the relationships between depression and the constructs.

As in optimism, the association between depression and hope finds support in the literature. Clinical trials with the elderly carried out interventions addressing hope and pointed to an improvement in life satisfaction and a significant reduction of the elderly’s depression (Clegg et al., 2014; Mirbagher et al., 2016; Mozooni et al., 2017). In aging, the loss of hope often occurs due to several geriatric syndromes, in addition to the limitations and changes that the body presents, and hope is what leads the elderly to find and have self-care, taking advantage of life with a better vision of future (Bahmani et al., 2016). The hope helps in the conservation of the health of the elderly in a vulnerable situation, explaining 64.9% of health conservation according to the study of Sung et al. (2017). In the present study, hope had a direct negative relationship with depression and a relationship mediated also by spiritual well-being. It still has connections with several other constructs and seems to mediate many relationships, influencing with greater strength in self-esteem.

Knowledge of the relationship of hope to depression is not new. Lack of hope is included as a diagnostic criterion for depression in DSM-V (2013), and, as a result, the relationship found in the present study was expected. Fehring et al. (1997) study indicated that significantly higher levels of hope and positive mood existed in elderly patients with high levels of intrinsic religiosity and spiritual well-being, with negative relationships with depression in the elderly with cancer. However, the possible mediations between them are not described in the literature, and the direct relation is still more accepted.

In addition to the studied constructs, it is important to note that among the demographic variables evaluated, the network analysis indicates that social class showed positive correlations with depression, indicating that higher incomes are associated with higher depressive indexes. Network analysis finds a direct negative relationship between social class, optimism, and spiritual well-being with degree of depression. No studies have been found in the literature that relate high social class with higher risk of depression. The studies generally present the opposite, that is, greater risks of depression in individuals with lower purchasing power (Kim et al., 2013, Gero et al., 2017).

Depression in the aging of the individual with higher social class can be seen as the expression of the abrupt decrease of social participation and social and cultural activities experienced by a senior of the upper classes throughout his life. The elderly of the lower classes do not feel as much a reduction in their activities because they have not experienced such great opportunities for social activities as cinema, theater, and travel.

The present study allows a comparison of the association between the constructs of Positive Psychology and depression, facilitating the choice of effective and preventive approaches to intervention with this population. This study shows the importance of the promotion and prevention of the health elderly. It also presents a view of network analysis and possible mediation in the relationships between depression and the studied constructs.

The study design also does not allow a cause-and-effect relationship between the constructs studied and depression, and it is limited to explore the associations between them. Individuals were no compared by time of use of antidepressant or time of follow-up in the outpatient setting but only by the severity/degrees of the disease.

## Conclusion

Higher degrees of depression are associated with an increase in negative affect and a decrease in positive affect, as well as a decrease in construct scores of spiritual well-being, social support, self-esteem, life satisfaction, positive affect, optimism, and hope. The relationships between depression and life satisfaction are mediated by optimism. Spiritual well-being, optimism, and depression had greater influence on a network of regular correlations between constructs. Depression presented a higher magnitude relationship in this network, followed by the constructs of dispositional hope and self-esteem, indicating that the three had a stronger impact on the network of constructs. Social class had a positive association with depression, which was unpublished in the literature consulted. The constructs that are strongly and indirectly related to depression are optimism, spiritual well-being, positive affect, and dispositional hope. The findings of this study aim to direct the scientific community in the search for interventions that will be more effective for the depressive patient and for the prevention of this psychopathology in the elderly population. Strategies for therapeutic intervention for the elderly with MDD should focus on spiritual well-being, dispositional hope, affection, and optimism, because that is greater chances of achieving a direct improvement in depression scores.

## Abbreviations

ADHS: Adult Dispositional Hope Scale
BDI-II: Beck Depression Inventory-II
EBIC: Extended Bayesian criterion
GDS: Geriatric Depression Scale
GLASSO: Graphical least absolutes shrinkage and selection operator
LSE: Life satisfaction scale
LOT-R: Revised Life Orientation Test
MMD: Major depressive disorder
MOS: Medical outcomes study social support scale
MSME: Mental state mini-exam
PANAS: Positive and Negative Affect Schedule
RSS: Rosenberg Self-Esteem Scale
SSRS: Spirituality Self-Rating Scale
